# Did imports of sweetened beverages to Pacific Island countries increase between 2000 and 2015?

**DOI:** 10.1186/s40795-021-00416-4

**Published:** 2021-05-20

**Authors:** Veronica Yueh Torng Lo, Gary Sacks, Emma Gearon, Colin Bell

**Affiliations:** 1 Australian Bureau of Statistics, National Data Acquisition Centre, Melbourne, Australia; 2 Deakin University, Institute for Health Transformation, Global Obesity Centre, Geelong, Australia; 3 Monash Department of Clinical Epidemiology, Cabrini Institute, Malvern, Australia; 4 Department of Epidemiology and Preventive Medicine, School of Public Health and Preventive Medicine, Monash University, Melbourne, Australia

**Keywords:** Imports, Pacific Island countries, Sugar sweetened beverage, Taxes, Trade, UN Comtrade, Fiji, Tonga

## Abstract

**Background:**

Nutrition-related chronic diseases are the major cause of illness and death in Pacific Island countries. Imports of sweetened beverages (SBs) are likely to be contributing but there is limited analysis of the quantities imported or the source countries of such beverages. The purpose of this study was to describe trends in the amount and types of SBs imported to Pacific Island countries and the impact of SB taxes on imports in Fiji and Tonga.

**Methods:**

A repository of official international trade statistics was used to collect data on the volume, dollar value and source countries of SBs exported to Pacific Island countries from 2000 to 2015. Corresponding population data was sourced from the Secretariat of the Pacific Community for per capita analyses. We also explored which countries earned the most from exporting SBs to the Pacific. Descriptive and regression analyses were used to describe trends over time for each country and for the region as a whole.

**Results:**

Imports of SBs to Pacific Island Countries from 2000 to 2015 increased by an average of 0.30 kg per person per year (*p* < 0.001). New Zealand and the USA were the largest income earners from SB exports to the Pacific over this period. The introduction of a tax did not impact the volume of SBs imported to Fiji. More data is needed to assess the impact of SBs tax on imports in Tonga.

**Conclusions:**

Exports of SBs to Pacific Island countries are increasing. Both importing and exporting countries should consider the health implications of trade in these products.

## Background

In recent years, Pacific Island countries have experienced a rise in non-communicable diseases (NCDs). The region has some of the highest prevalence rates of type 2 diabetes (47%) and obesity (75%) in the world [1]. Moreover, 60 to 77% of total deaths in Pacific Island countries are attributable to NCDs [1].

Globalisation, specifically the global trade of foods, may contribute to NCDs [2]. While trade has improved food security in developing countries, importation of processed foods and beverages that are nutrient-poor and/or energy dense such as soft drinks and instant noodles can contribute to unhealthy diets. This is particularly evident in Pacific Island countries where domestically produced foods low in fat and high in complex carbohydrate, dietary fibre, and foods of plant origin [3], have been largely replaced with imported, processed foods [4]. In Palau, for example 84% of food supplies are imported [5]. This has led to a nutrition transition marked by increased availability of imported foods such as rice and bread and decreased consumption of local food such as taro and yam [1]. Seafoods have been replaced by imported meats high in fat and fruit and starches have been replaced by sugar and confectioneries [1, 6]. Such dependence on imported foods, associated declines in domestic production, economic shocks and climate change are posing new threats to food security in the Pacific and contributing to NCDs [7].

Sugar sweetened beverages are defined as beverages that contain added sugars, or are a significant source of free sugars, such as soft drinks, energy drinks, juices and milk drinks. A study conducted in 2011 revealed high soft drink consumption, with 31 l (L) being consumed per person in Tonga in 2011 and 84 L being consumed per person in Palau that same year [8]. In Tokelau, increased sugar availability has been associated with dental caries in children [9]. Between 1963 and 1999, sugar imports increased eight times and mean number of decayed and filled teeth increased from 3 to 5 [9].

Despite high rates of NCDs in the Pacific and our knowledge of the contribution of foods/beverages high in fat, sugar, and salt to NCDs, including caries, little research has been done to investigate volumes of unhealthy products imported to Pacific countries. International Trade Databases document the flow of goods, including foods, between countries and tapping into this information may help policy makers identify import trends detrimental to health.

The aim of this paper was to track imports of SBs to Pacific Island countries over time and identify source countries and related earnings. We also investigated the extent to which SBs imports decreased in Fiji and Tonga following the introduction of taxes in those countries.

## Methods

### Study design

We conducted a secondary analysis of publicly available food commodities data to describe changes in imports of sweetened beverages to Pacific Island countries between 2000 and 2015.

### Data sources

The United Nations Commodity Trade Statistics Database (UN Comtrade) was used to collect information on the types and volume (or weight) of SBs imported to Pacific Island countries, dollar value (in US dollars) and source countries from 2000 to 2015. The UN Comtrade has imports and exports information reported by statistical authorities from approximately two hundred countries or areas. It contains trade data from 1962 to the most recent year [10].

Pacific Island countries’ population data from year 2000 to 2015 was sourced from the Statistics for Development Division (SDD) of the Secretariat for the Pacific Community (SPC) and summed to give an estimate of the total population across all countries (Table 4 in Appendix 1) [11]. Where data was missing for a country in a particular year, the missing data was inferred using linear regression analysis of all available data points (between 2000 and 2015).

### Countries

We focused on 12 countries that are members of the Pacific Island Forum Secretariat (PIFS), hereafter referred to as Pacific Island countries (PICs). These included Cook Islands, Federated States of Micronesia, Fiji, French Polynesia, Kiribati, New Caledonia, Palau, Samoa, Solomon Islands, Tonga, Tuvalu, and Vanuatu. Republic of Marshall Islands, Nauru, and Niue are also members of PIFS but import data were not available. Fiji and Tonga introduced SB taxes during the study period (Table 1). Average GDP for the12 included Pacific Island countries increased from USD 3.4 billion in 2000 to USD 8.9 billion in 2015 [12]. In 2015, GDP in Fiji was USD 4.6 billion and Tonga was USD 0.4 billion [12].

**Table 1:** SB taxes introduced in Fiji and Tonga between 2000 and 2015

COUNTRY	YEAR OF ADOPTION	IMPORT TARIFF RATE	EXCISE TAX RATE	SBs TAXED
**Fiji**	**2011**	No specific tariff^a^ 32% import duty applied to beverages whether or not they are sweetened	No specific tax 15% excise tax applied to beverages whether or not they are sweetened	Soft drinks and juice, whether or not sweetened, excludes sweetened milk (HS22.02)
**Tonga** ^b^	**2013**	No specific tariff Average import tariff of 10% on food and beverages	T 0.50 /L, replacing an existing 15% import duty	Sweetened beverages, including flavoured milk (HS22.02)

^a^ No specific tax means that the rate of taxation for SBs was no greater than that of other categories of food or drink [13]

^b^ Tonga doubled the excise tax on SBs in 2016 to T$1/L (USD 0.43) and there was a further increase to T$1.50/L (USD 0.65) in 2017 and changes to broaden the tax to include fruit juices and powdered drinks

### Sweetened beverages

We used the World Health Organization (WHO) nutrient profile model for the Western Pacific region to define SBs for this study [14]. This model was designed to assist countries in making decisions about appropriate marketing of food and beverages to children. It provides nutrient cut-points for sugar, salt, and saturated fat in 18 food categories above which it is not recommended that foods be advertised to children. The model uses the Harmonized Commodity Description and Coding System (HS code), an international standardised system of names and numbers for the classification of commodities, which is used by UN Comtrade. We defined SBs as HS Codes 20.09 (juices), 04.02 (milk drinks), 21.01.12 (tea and coffee), 22.02 (water, including mineral and aerated drinks) (Table 2; Table 5 in Appendix 2).

**Table 2:** Sweetened beverage HS codes and description of the beverages they contain from the WHO Nutrient Profile Model

HS Codes^a^	Description of beverages
**JUICES**	
**20.09**	Fruit juices (including grape must)^b^ and vegetable juices, unfermented and not containing added spirit, whether or not containing added sugar or other sweetening matter
**MILK DRINKS**	
**04.02**	Milk and cream, concentrated or containing added sugar or other sweetening matter
**TEA & COFFEE**	
**21.01.12**	Preparations with a basis of extracts, essences, or concentrates with a basis of coffee
**WATER, INCLUDING MINERAL AND AERATED DRINKS**	
**22.02**	Waters, including mineral and aerated, containing added sugar or other sweetening matter or flavoured. Soft drinks are included in this category.

^a^HS Codes is an international standardised system of names and numbers for the classification of commodities

^b^ Grape ‘must’ is freshly crushed fruit juice (usually grape) that contains the skins, seeds, and stems of the fruit

### Data analysis

We conducted a linear regression analysis to describe trends in SB imports in kilograms per person per year for all 12 Pacific Island countries from 2000 to 2015. Upon visual inspection of the data, it was evident that an unusually large amount of SSBs were imported to Pacific Island Countries in 2010. After exploring stockpiling, slumps in domestic production, an increase in tourism, cyclones, and other potential explanations with colleagues in the Pacific we could not find evidence to explain the 2010 observation, and so we treated this as an outlier and removed 2010 values from the primary analysis. Sensitivity analyses including data for 2010 were not appreciably different to the primary analyses (Table 6 in Appendix 3). Descriptive analyses were conducted to determine which countries were the top exporters of SB to Pacific Island countries based on earnings (US dollars). We used world consumer price inflation data (annual % from 2000 to 2015) from the World Bank to adjust earnings for inflation (base year = 2015). Descriptive analysis was also used to determine trends over time in types of SBs (juices, milk drinks, tea and coffee, other) imported. To see if imports decreased following the introduction of taxes, we also conducted linear regression analyses to describe trends in SBs imports in kilograms per person per year for Fiji and Tonga.

### Ethics

We applied for and were granted an exemption for ethics approval by the Deakin University Human Research Ethics Committee (2017–205).

## Results

### Change in SB imports over time

**Fig. 1:**
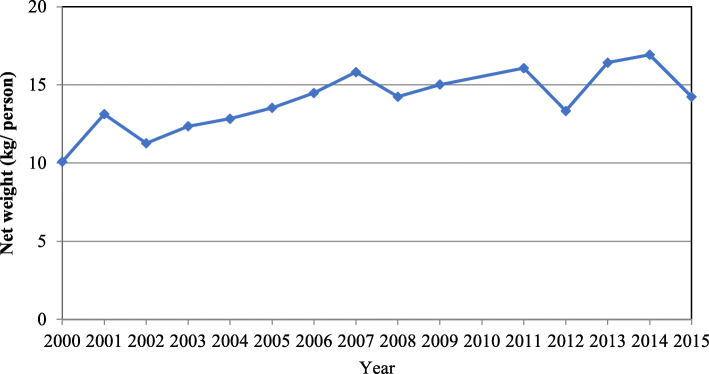
SBs (kg/person) imported to Pacific Island countries from 2000 to 2015

Imports of SBs to PICS increased from 24 million kg in 2000, to 39 million kg in 2015 (Fig. 1). Our regression analysis revealed a statistically significant increase of 0.30 kg/person per year (95% CI: 0.15, 0.45) of SBs to PICs – or 4.5 kg/person (95% CI: 2.25, 6.75) over the 15-year period.

### Trends in imports of SBs by sub-category

**Fig. 2:**
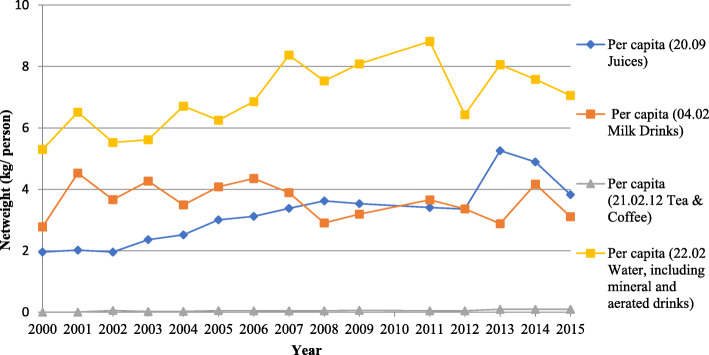
SBs imported to PICs by sub-category between 2000 and 2015

‘Water, including mineral and aerated drinks’ were imported in the highest volumes compared to other sub-categories (Fig. 2). Between 2000 and 2015, we observed significant increases in imports per person per year of ‘juices’ (HS Code: 20.09), which increased by 0.18 kg/person per year (95% CI: 0.12 to 0.23), ‘tea and coffee preparations’ (HS Code: 21.01.12), which increased by 0.01 kg/person per year (95% CI: 0.00, 0.01), and ‘water, including mineral and aerated drinks’ (HS Code: 22.02) which increased by 0.15 kg/person per year (95% CI: 0.05, 0.25). Imports of ‘milk drinks’ (HS Code: 04.02) per person per year did not significantly change over the period 2000 to 2015 (− 0.03 kg/person per year (95% CI: − 0.1, 0.03).

### Top exporter earners from SBs

The inflation adjusted total trade value of exports of SBs to the Pacific Islands was 1.1 billion in 2015 dollars, with New Zealand, USA and France being the top earning countries over this 15-year period (Table 3).

**Table 3:** Value of SB exports to Pacific Island Countries, 2000–2015 in 2015 dollars

Export Country	Trade Value (USD)^a^	Share (%)
New Zealand	197,514,296	18.4
USA	153,067,437	14.2
France	137,238,196	12.8
Australia	63,819,822	5.9
Fiji	22,192,803	2.1
Other countries	501,154,671	46.6
**Total**	1,074,987,225	100

^a^ Adjusted for annual world consumer price inflation

### SBs imports to Fiji and Tonga

**Fig. 3:**
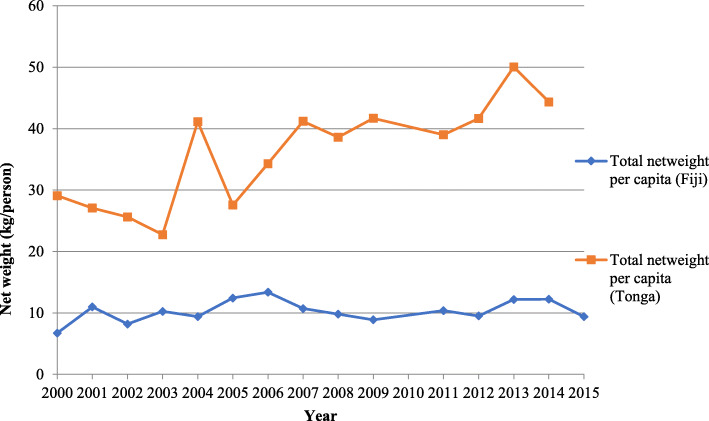
Imports of SBs (kg/person) to Fiji and Tonga from 2000 to 2015

We did not observe a statistically significant change in SB imports to Fiji between 2000 and 2015 (Fig. 3), average change of 0.11 kg/person per year (95% CI: −.09, 0.32). We did not observe an obvious downward trend post the introduction of the 2011 SB tax when visually inspecting the trend. We observed a statistically significant increase in SB imports to Tonga between 2000 and 2014, which increased by 1.50 kg/person per year (95% CI: 0.87, 2.14). It was not possible to draw conclusions on the effect of taxation in Tonga, given there was only one data point (2014) following the introduction of the tax.

## Discussion

The quantity of SBs imported to PICs increased significantly from 2000 to 2015 with exporting countries, particularly New Zealand, the USA and France, making a total of USD 1.1 billion from sales. ‘Water, including mineral and aerated drinks’ were the most common type of SBs imported, and imports of juices, milk drinks and ‘water, including mineral and aerated drinks’ all increased significantly between 2000 and 2015.

Liberalisation of trade barriers may be one explanation for the observed increase in SBs imports. A longitudinal analysis of 44 low- and middle- income countries describes trade liberalisation as a vector for sugar after observing lower tariffs translate into increased imports and increased sales over the 13 years from 2001 to 2014 [15]. Similarly, a study comparing sugar-sweetened carbonated beverage sales in Vietnam and the Philippines found growth in sales in Vietnam, led by foreign-owned companies, significantly accelerated after trade and investment liberalization [16]. Finally, a study in 11 PICs between 2003 and 2013 reported trade liberalisation had a positive and statistically significant effect on imports of processed foods to Pacific Island countries [17]. Our results are consistent with the study’s findings over the same period of time. In addition to detrimental impact of diets high in processed foods on nutrition and health [18], a reliance on imports also has the potential to undermine domestic production and local food systems and loss of traditional knowledge and biodiversity [19].

Another explanation may be an increase in advertising for SBs. A study investigating the effects of unhealthy food advertising on children and adolescents in Suva, Fiji demonstrated an impact on food preferences and requests [20]. A further potential explanation is an association with development assistance. High levels of development assistance have been associated with high levels of food imports from the same countries. A survey of the availability of imported foods in Pacific Island countries found that 56% of food items in Nauru’s stores were manufactured in Australia, a country that Nauru is heavily reliant on for aid [9]. Our data provides preliminary support for this contention given that New Zealand, Australia, France and the USA provide substantial development aid to Pacific Island countries, although more detailed analysis would be required to investigate it in a rigorous way.

This study addresses, in part, a lack of research evaluating the effectiveness of SB taxes in the Pacific Island region [21]. An assessment of the tax in Fiji revealed the cost of SBs increased in response to the tax [9]. It may be that the increase was not sufficient to impact imports, that a clear picture is being complicated by sizable domestic production of SBs in Fiji, or that our measure is too blunt to capture the impact of the tax on imports. In line with the decrease we observed in the imports of SBs to Tonga between 2013 (when the tax was introduced) and 2014, using a time series analysis, Teng et al. reported that successive tax increases from 2013 in Tonga were associated with increased prices, decreased taxed beverage imports, and increased locally bottled water [22].

### Strengths and limitations

Strengths include the secondary analysis of publicly available commodities data for answering health related questions. We were also able to take advantage of natural experiments and track the impact of new SB taxes in Fiji and Tonga on levels of imported SBs. The UN Comtrade database has the sole purpose of providing trade data. However, it is not detailed enough to differentiate between particular types of SBs. For example, we were unable to exclude artificially sweetened beverages or bottled water from HS code 22.02. The proportion of water-based beverage imports to the Pacific that are artificially sweetened is not known but for comparison, it is estimated that non-sugar sweetened beverages comprised 36% of water-based beverage sales in Australia in 1997 and 59% in 2018 [23]. Also, it is continuously updated which means it may yield different data with successive data extractions. It should be noted that taxes in Fiji and Tonga apply to both sugar-sweetened and non-sugar sweetened beverages. Another limitation is the lack of information on domestic production which meant we were not able to fully quantify availability of SBs in some countries. Tonga has limited domestic production of SBs but Fiji has a Coca Cola Amatil factory that not only produces SBs for Fiji but also a number of other Pacific countries (see Table 3) [17]. A further limitation is that our population level analyses were not able to account for tourists who visit Pacific countries in large numbers and consume at least some of the imported SBs.

## Conclusions

These results may help policy makers in the Pacific Island countries assess whether controls are needed on SB imports. Also, the results draw attention to the fact that tax payers in New Zealand and Australia are paying for the increasing costs of NCDs in the PICs (through aid funding provided to these countries) on the one hand while, on the other, companies based in New Zealand and Australia are profiting from exporting SBs to the same countries. For example, Tonga received AUD32.9 million (~ USD 23.5 million) worth of aid from Australia in 2011–2012 [24]. In spite of the noted limitations of the UN Comtrade Database, its ability to shed light on imports of SBs to Pacific Island countries may have value for determining availability of other foods or food-groups contributing to NCDs.

## Data Availability

Public access to the UN Comtrade database is available here. Public access to Population data for Pacific countries is available here. Our analysis of the data is available as supplementary material.

## Appendix 1

**Table 4:** Population data for all Pacific Island Countries of Interest

**Year**	**Fiji**	**Tonga**	**Tuvalu**	**Vanuatu**	**Samoa**	**Solomon Islands**	**Cook Islands**
2000	798,751	99,162	9540	189,542	175,066	416,018	15,743
2001	804,572	99,755	9576	194,605	176,710	427,804	15,030
2002	810,335	100,238	9561	199,750	177,751	439,987	15,113
2003	816,029	100,741	9682	204,985	178,683	452,555	15,193
2004	821,637	101,265	9980	210,319	179,501	465,494	15,270
2005	827,125	101,482	10,285	215,769	180,203	478,792	15,345
2006	832,449	101,991	10,432	221,344	180,741	492,438	15,324
2007	837,271	102,248	11,130	227,056	181,267	506,422	15,369
2008	840,032	102,652	11,035	232,908	181,964	520,617	15,426
2009	843,888	103,023	11,093	234,023	182,578	515,870	15,479
2010^c^	847,793	103,365	11,149	245,376	183,123	539,469	15,529
2011	851,744	103,036	11,206	251,784	187,820	553,254	14,974
2012	*856,571*	103,192	*11,183*	*255,678*	*187,812*	*562,307*	*15,964*
2013	859,200	103,300	10,900	264,700	192,203	615,804	*16,049*
2014	*865,602*	*103,131*	*11,449*	*266,905*	*189,991*	*586,379*	*16,134*
2015	867,000	103,300	11,300	277,600	187,300	515,870	*16,219*
2016^a^		100,651	11,534	272,459	195,979	651,700	17,459
**Year**	**Federated States of Micronesia**	**French Polynesia**	**Kiribati**	**New Caledonia**	**Palau**	**Total Population**^**b**^	
2000	107,021	*259,530*	84,230	*208,708*	19,129	2,382,440	
2001	106,840	*260,190*	85,872	*212,825*	19,293	2,413,072	
2002	106,612	*260,850*	87,396	*216,941*	19,454	2,443,988	
2003	106,339	*261,510*	88,756	*221,058*	19,610	2,475,141	
2004	106,021	*262,170*	90,272	*225,174*	19,761	2,506,864	
2005	105,654	*262,830*	92,533	*229,291*	19,907	2,539,216	
2006	105,232	*263,490*	93,698	*233,407*	20,047	2,570,593	
2007	104,754	*264,150*	95,470	*237,524*	20,162	2,602,823	
2008	104,217	*264,810*	97,201	*241,640*	20,278	2,632,780	
2009	103,620	*265,470*	98,989	*245,757*	20,397	2,640,187	
2010^a^	102,843	*266,130*	103,058	*249,873*	20,518	2,688,226	
2011	*103,928*	*266,790*	*103,975*	*253,990*	20,643	2,723,144	
2012	*103,665*	*267,450*	*105,865*	*258,106*	17,501	2,745,294	
2013	103,395	269,100	108,800	262,223	17,800	2,823,474	
2014	*103,140*	270,500	*109,645*	268,767	17,588	2,809,232	
2015	102,300	263,000	110,136	265,600	17,661	2,737,286	
2016^c^	104,600	273,800	115,300	277,000	17,800	2,912,915	

^a^Data for the year 2016 was in linear trend analyses to make inferences about missing population data. Cells in which the population data for a specific year was inferred are presented in *italics*

^b^ Total population data represents the sum of all 12 countries.

^c^ Data for the year 2010 was excluded from the primary analysis

## Appendix 2

**Table 5:** SSB import data for PICs from 2000 to 2015

Year	TOTAL NETWEIGHT JUICES (20.09)	TOTAL NETWEIGHT MILK DRINKS (04.02)	TOTAL NETWEIGHT TEA AND COFFEE (21.01.12)	TOTAL NETWEIGHT WATER, INCLUDING MINERAL AND AERATED DRINKS (22.02)	TOTAL NETWEIGHT OF SB IMPORTS (KG)	NETWEIGHT OF SB IMPORTS TO FIJI (KG)	NETWEIGHT OF SB IMPORTS TO TONGA (KG)
2000	4,672,566	6,629,494	1290	12,630,011	24,025,113	5,364,492	2,884,047
2001	4,884,104	10,932,450	7200	15,716,764	31,688,888	8,842,313	2,700,199
2002	4,781,340	8,954,690	132,949	13,506,675	27,535,113	6,649,207	2,568,555
2003	5,838,002	10,569,714	54,201	13,888,543	30,560,474	8,375,517	2,291,880
2004	6,311,478	8,757,039	69,087	16,817,855	32,182,719	7,733,936	4,164,337
2005	7,637,703	10,362,393	124,066	15,877,264	34,344,225	10,290,525	2,796,673
2006	8,017,202	11,195,688	119,095	17,632,453	37,236,289	11,137,006	3,497,240
2007	8,795,573	10,131,184	109,732	21,786,451	41,169,794	8,977,592	4,211,572
2008	9,543,735	7,653,258	123,814	19,827,138	37,499,351	8,247,607	3,961,497
2009	9,322,623	8,436,547	168,002	21,348,118	39,648,951	7,510,230	4,293,835
2010^a^	30,603,496	9,236,683	146,195	26,106,970	66,423,182	29,323,873	4,412,428
2011	9,287,323	9,959,137	124,675	24,018,380	43,808,565	8,846,359	4,018,894
2012	9,220,650	9,227,892	122,418	17,669,660	36,616,978	8,144,018	4,297,561
2013	14,848,826	8,135,919	275,940	22,752,183	46,367,487	10,481,273	5,168,096
2014	13,746,041	11,726,753	261,336	21,310,072	47,573,716	10,599,359	4,573,153
2015	10,484,041	8,508,732	256,743	19,323,864	38,966,734	8,154,759	NA

^a^ Data for the year 2010 was excluded from the primary analysis

## Appendix 3

**Table 6:** Comparison of output for primary and sensitivity analyses

	Primary Analyses (excluding data for the year 2010)		Sensitivity Analyses (including data for the year 2010)	
**TOTAL SSB IMPORTS PER CAPITA**				
Increase per person per year	0.30 (0.15, 0.45)	*p*-value < 0.001	0.38 (0.05, 0.70)	*p*-value =0.027
Increase per person over 15 years	4.50 (2.25, 6.75)		5.70 (0.75, 10.50)	
**IMPORTS OF JUICES (20.09) PER CAPITA**				
Increase per person per year	0.18 (0.12, 0.23)	*p*-value < 0.001	0.24 (0.00, 0.47)	*p*-value =0.050
Increase per person over 15 years	2.70 (1.80, 3.45)		3.60 (0.00, 7.05)	
**IMPORTS OF MILK DRINKS (04.02) PER CAPITA**				
Increase per person per year	−0.03 (− 0.10, 0.03)	*p*-value =0.311	− 0.03 (− 0.10, 0.03)	*p*-value =0.279
Increase per person over 15 years	− 0.45 (− 1.50, 0.45)		− 0.45 (− 1.50, 0.45)	
**IMPORTS OF TEA AND COFFEE (21.01.12) PER CAPITA**				
Increase per person per year	0.01 (0.00, 0.01)	*p*-value < 0.001	0.01 (0.00, 0.01)	*p*-value < 0.001
Increase per person over 15 years	0.15 (0.00, 0.15)		0.15 (0.00, 0.15)	
**IMPORTS OF WATER, INCLUDING MINERAL AND AERATED DRINKS (22.02) PER CAPITA**				
Increase per person per year	0.15 (0.05, 0.25)	*p*-value =0.008	0.16 (0.04, 0.28)	*p*-value =0.010
Increase per person over 15 years	2.25 (0.75, 3.75)		2.40 (0.60, 4.20)	
**Total IMPORTS OF SSBS TO FIJI PER CAPITA**				
Increase per person per year	0.11 (−0.09, 0.32)	*p*-value =0.256	0.29 (− 0.45, 1.03)	*p*-value =0.416
Increase per person over 15 years	1.65 (−1.35, 4.8)		4.35 (−6.75, 15.45)	
**Total IMPORTS OF SSBS TO TONGA PER CAPITA**				
Increase per person per year	1.50 (0.87, 2.14)	*p*-value =0.0002	1.52 (0.93, 2.12)	*p*-value =0.000095
Increase per person over 14 years	21.00 (12.18, 29.96)		21.28 (13.02, 29.68)	

## Abbreviations

AUD: Australian Dollar
CI: Confidence interval
GACD: Global Alliance for Chronic Diseases
GDP: Gross Domestic Product
HS: Harmonised System or Harmonised Commodity Description and Coding System
NCD: Non-communicable disease
NHMRC: National Health and Medical Research Council of Australia
PIC: Pacific Island Countries
PIFS: Pacific Islands Forum Secretariat
SDD: Statistics for Development Division of the Secretariat of the Pacific Community
SB: Sweetened beverages
SPC: Secretariat of the Pacific Community
UN Comtrade: United Nations Commodity Trade Statistics Database
USA: United States of America
USD: United States Dollar
WHO: World Health Organization
